# An Approach to Aligning Categorical and Continuous Time Series for Studying the Dynamics of Complex Human Behavior

**DOI:** 10.3389/fpsyg.2021.614431

**Published:** 2021-04-16

**Authors:** Kentaro Kodama, Daichi Shimizu, Rick Dale, Kazuki Sekine

**Affiliations:** ^1^University Education Center, Tokyo Metropolitan University, Tokyo, Japan; ^2^Department of Integrated Educational Sciences, Graduate School of Education, University of Tokyo, Tokyo, Japan; ^3^Department of Communication, University of California, Los Angeles, Los Angeles, CA, United States; ^4^Faculty of Human Sciences, Waseda University, Saitama, Japan

**Keywords:** visualization, quantification, recurrence analysis, speech-motion coupling, rap

## Abstract

An emerging perspective on human cognition and performance sees it as a kind of self-organizing phenomenon involving dynamic coordination across the body, brain and environment. Measuring this coordination faces a major challenge. Time series obtained from such cognitive, behavioral, and physiological coordination are often complicated in terms of non-stationarity and non-linearity, and in terms of continuous vs. categorical scales. Researchers have proposed several analytical tools and frameworks. One method designed to overcome these complexities is recurrence quantification analysis, developed in the study of non-linear dynamics. It has been applied in various domains, including linguistic (categorical) data or motion (continuous) data. However, most previous studies have applied recurrence methods individually to categorical or continuous data. To understand how complex coordination works, an integration of these types of behavior is needed. We aimed to integrate these methods to investigate the relationship between language (categorical) and motion (continuous) directly. To do so, we added temporal information (a time stamp) to categorical data (i.e., language), and applied joint recurrence analysis methods to visualize and quantify speech-motion coordination coupling during a rap performance. We illustrate how new dynamic methods may capture this coordination in a small case-study design on this expert rap performance. We describe a case study suggesting this kind of dynamic analysis holds promise, and end by discussing the theoretical implications of studying complex performances of this kind as a dynamic, coordinated phenomenon.

## Introduction

Recent theoretical and empirical work in cognitive science has argued that we cannot separate cognition from the body and its environment. In any complex performance, these dimensions are interdependent (e.g., Anderson et al., 2012; Riley et al., 2012). This notion is called embodiment, or situated cognition (Chemero, 2009). From the viewpoint of embodiment, cognitive processes related to language and communication interact with bodily motion and behavior (e.g., Glenberg and Kaschak, 2002; Clark, 2006; Richardson et al., 2008; Chemero, 2009; Shockley et al., 2009; Zwaan, 2014). We can thus consider language specifically, and cognition in general, as sustained by a complex pattern of interaction among brains, bodies and environments (see recent review and discussion in Favela and Chemero, 2015).

The role of gesture in communication illustrates these theoretical ideas. Research in this area has shown that the body is not only connected to cognitive processes, but also to linguistic processes (Kita and Özyürek, 2003). Since McNeill (1992) found the significant relationships between gestures and speech, both in production and comprehension, the number of studies on speech and gesture has increased. Previous research has shown that gestures facilitate the speaker’s speech process, and evidence for intrapersonal synchronization of speech rhythms and hand movements (Chui, 2005). For example, when participants were asked to not move their hands while speaking, the proportion of unfilled pauses (Graham and Heywood, 1975) or fillers (Rauscher et al., 1996) increased. These findings suggest that speech is closely linked to meaningful hand movements.

A fundamental open challenge in this area is to understand how complex human performances, such as musical or linguistic performances, involve coordination among very different measurements. For example, language is often measured at an abstract (categorical) level, focusing on verbal forms described as symbols ordered in time. Bodily movement, whether in dance or other musical performance, can often be measured using automatic tracking or analysis, granting researchers scalar (continuous) time series (for review see Richardson et al., 2014). A common technique to link these types of measurements is to aggregate movement data and superimpose them on language categories (e.g., words, sentences) to explore their relationships (a “temporally aggregative” method: Coco and Dale, 2014). An alternative approach would be to preserve the dynamic qualities of each type of measure, and use new analytic tools that can link these dynamics. But this poses a challenge—language and body are often measured at different timescales (one abstract, word based; the other with continual sampling). In this short paper, we summarize a technique that can integrate language data with continuous body motion data and shed some light on how they are coordinated. We then showcase a small-scale case study of expert lyrical performance, in particular an expert rap performance, to demonstrate the promise this method may hold for future research. We end by summarizing future applications of this method, and its promise for contributing to new theoretical developments in various domains of human performance.

We look to methodologies developed under a particular theoretical framework, one designed to embrace the dynamic nature of human cognition and performance. The dynamical systems approach (DSA) has been widely applied to human movement science, developmental psychology, and cognitive science (Chemero, 2009; Richardson et al., 2014). Compared to a traditional approach, which focuses on *internalized* computation in the brain, DSA focuses more on interactions among the body (including the brain), environment, and task. The DSA has provided both a theoretical framework and analytical tools based on the study of non-linear dynamical systems (e.g., Van Orden and Riley, 2005). In this domain, a particular methodology called *recurrence quantification analysis* (RQA) has been used to flexibly analyze dynamic measurements of all sorts. It has few statistical assumptions, and few constraints on the kind of time series that is analyzed (Zbilut and Webber, 1992). For this reason, it seems especially well suited to dynamically integrated distinct types of temporal data.

RQA is based on quantifying a visualization called the recurrence plot (RP). A RP is a two-dimensional graph visualizing recurring patterns of a dynamical system. The plot is essentially a matrix of (*i*, *j*) entries in which these matrix elements correspond to those times *i* and *j* at which a state of a dynamical system recurs in the space of its overall behaviors (Marwan et al., 2007). This recurrence (or “revisitation”) is assessed numerically by comparing a time series to itself over time. Consider the idea of human dance or other performance. At a given time *i* the human body will be in a particular configuration. If at time *j* the human body is in a similar configuration (e.g., measured with Euclidean distance), then we count (*i*, *j*) as a point on the RP. The overall RP is thus a visualization of the tendency for a system to “come back” to its various positions over time. It is an advanced technique of non-linear data analysis and was originally developed in the fields of descriptive statistics and chaos theory (Eckmann et al., 1987) and promptly thereafter applied to physiological signals (Webber and Zbilut, 1994).

RQA is a suite of tools for quantifying the extent and duration of these recurrences in the RP system (Marwan et al., 2007). It was originally developed to uncover subtle time correlations and repetitions of patterns. RQA can provide researchers with some useful measures to quantify self-organizing dynamical system behavior. For example, it can capture how deterministic or periodic a process is by the orderliness of how points (*i*, *j*) fall on straight lines. These lines reflect sequences of revisited behavior. In contrast, RPs with very little structure, and the appearance of “TV noise,” reflect a dynamical system that is behaving in a random or unstructured way.

RP and RQA have been applied to both continuous data (for example, a numeric value obtained by sensor devices) and categorical data (for example, a letter or word sequence in literature pieces) (Coco and Dale, 2014). Louwerse et al. (2012) applied Categorical RQA to both verbal and non-verbal behaviors (Louwerse et al., 2012). They categorized verbal data like dialog moves and the words they contain, and also categorized non-verbal data such as gesture and facial expression by coding them into discrete categories (e.g., iconic gesture or smile). For such categorical or coded data, they added temporal information (i.e., time stamp) at 4 Hz and applied Categorical RQA. Although they addressed with communication behavior including verbal data, they did not use continuous data or integrate categorical data with continuous data. Walton et al. (2018) applied Categorical RQA to musical performance data (Walton et al., 2018). They generated categorical time series of the key and press timing pressed by keyboard players using the MIDI device and added temporal information (i.e., time stamp) at 96 Hz. Although they applied Categorical RQA for these musical performance data and Continuous RQA for body movement data, they did not integrate them.

These previous studies are important and suggestive in terms of extending Categorical RQA by adding temporal information. However, most previous studies have applied these recurrence methods (categorical or continuous) separately. We aimed to integrate the two different types of information within the same recurrence analytical framework in order to visualize and quantify such different kind of time series data like verbal (categorical) and non-verbal (continuous) behaviors (e.g., speech-action coordination/coupling).

For this purpose, we developed the categorical recurrence analysis and applied the joint recurrence analysis methods (see “Data Analysis” section under “Materials and Methods”). If these can be integrated within the same analytical framework, recurrence analysis can be extended widely to visualize and quantify various complex phenomena in cognitive science. As a first attempt to explore such a possibility, the current pilot study focused on a speech-motion coordination/coupling during a rap performance. Because rap or hip-hop music has a relatively obvious rhythm structure, and because mind-body coordination/coupling is important in rapping behavior, we assumed that this relationship would be relatively easy to extract using recurrence methods. Our single-case study thus illustrates the feasibility of this alignment of time series types.

## Materials and Methods

### Participant

A professional rapper (Japanese male, 30 years old, right-handed, native language is Japanese) participated in our experiment. He has more than 15 years of rapping experience and was the champion of a national freestyle rap battle. He has also released his recordings as a professional musician. The participant signed an informed consent form, agreeing to participate in this study.

### Apparatus

**FIGURE 1:**
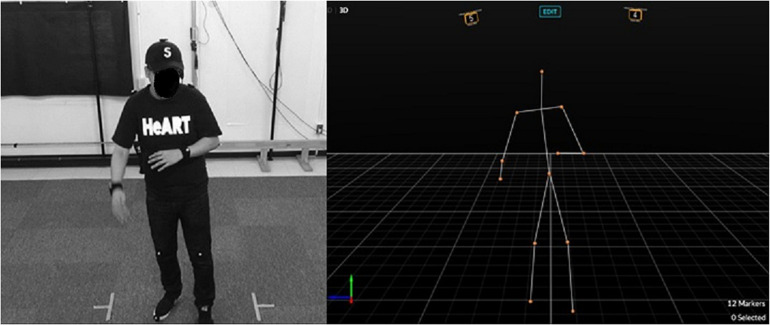
An experimental situation and the motion capture system (from Kodama et al., 2019).

We used a 3D motion capture system (OptiTrackFlex13, Natural Point, Inc.) to measure the participant’s body movements (sampling frequency was 120 Hz) (Figure 1). Twelve reflective markers were attached to the participant’s body (head, both shoulders, both elbows, both wrists, hip, both knees, and both toes). We used Motive (Natural Point) to process the time series data, MATLAB (R2017b, MathWorks) and RStudio (1.1.423) to analyze the data. We also used a video camera (HDR-PJ720, Sony) (frame rate of 50 FPS) and a headset microphone (Hafone) (sampling rate of 44 kHz). To analyze the audio data, we used Audiacity (2.2.2) after down-sampling at 25 FPS.

### Procedure

We required a professional rapper to perform parts of a rap song in Japanese, which included an Introduction, Verse, and Hook. This performance provided data for the analysis, and totalled approximately 1 min. Before recording, we attached 12 reflective markers and a microphone to his body, and we asked him to stand in front of the camera. We then instructed him to perform naturally, as if he were presenting a live performance. After sound checking, we started the recording. In this paper, we report the results of our analysis of part of that segment of his performance (from the first Verse and Hook).

### Data Analysis

To visualize and quantify the rhythmic structure and coordinated behavior between the rap (speech) and body movement (motion), we applied *recurrence analyses* (for tutorials, refer to Webber and Zbilut, 2005; Wallot, 2017). We briefly describe these recurrence methods, and we introduce the *joint recurrence method* (Marwan et al., 2007) to integrate them as described in the following paragraph.

In the case of continuous data, time series data are embedded, their trajectory is reconstructed in a higher dimensional phase space, and the distances between all possible combinations of each vector are calculated and distributed within a distance matrix (Webber and Zbilut, 2005). All elements in the distance matrix with distances at or below a threshold (i.e., radius) are said to be *recurrent* (recurrence point) and are included in the recurrence matrix, while all other elements are excluded from it. Such calculations and definitions are used to construct a *recurrence plot* (RP), a method of visualization that shows the dynamic properties and temporal patterns of the system as a two-dimensional representation (Eckmann et al., 1987). A *recurrence quantification analysis* (RQA) allows researchers to quantify and assess the properties of a dynamical system, based on RP or the phase space trajectory (more detail in Webber and Zbilut, 2005). This study reported four of the most common RQA measures, namely, the *recurrencerate* (*RR*), *percent determinism* (*DET*), *maxline* (*maxL*) and *mean line* (*L*). *RR* is the density (percentage) of recurrence points in a RP; *DET* is the percentage of recurrence points forming diagonal lines in the recurrence plot given a minimal length threshold; *maxL* is the length of the longest diagonal line; *L* is the average of the diagonal line’s length (Coco and Dale, 2014). The units of these lines are indicated in time (e.g., seconds). If the length of these lines is long, it means that the system repeats the same state persistently for a long time. These measures have been interpreted as indexes related to stability or complexity of human motor/posture systems (e.g., Riley et al., 1999; Pellecchia et al., 2005).

In this study, we used only the hip and right wrist movements data in a vertical direction as continuous data, because the hip position can be considered as a collective index of whole-body movement at the macro scale (Shockley et al., 2003) and the right hand movement can be interpreted as a specific index of rap-related rhythmic movement at the micro scale (i.e., gestures: Almeida and Sousa, 2019). After each time series was smoothed, it was then downsampled at 25 Hz to integrate it with the categorical data.

In the case of categorical data, researchers generally need not embed the data in a phase space, but to define the level or unit of analysis (e.g., a word or letter). Each unit is converted into numeric categorical sequence (e.g., *a*→1, *b*→2, *c*→3, …). Researchers can create a recurrence point when the two series (original and self-copied sequential series) share the same state (i.e., the same word/letter) in time. Thus, the same RQA measures can be calculated and they provide meaningful indexes that can be considered *dynamic natural language processing*. For example, determinism (*DET*) reflects how sequences of behaviors are repeating, and so relates to a data set’s compressibility ratio*; recurrence rate* (*RR*) reflects how often single behaviors are repeating, and so is related to *co-occurrence* (see Dale et al., 2018).

We obtained sequential data by analyzing the lyrics and converting each voice unit into a Japanese vowel (*a/i/u/e/o*), a syllabic nasal (*n*), or an assimilated sound (*x*). We chose a vowel as a main unit of analysis, because rap lyrics tend to rhyme (match rhyming words at vowel level) more often in hip-hop music, generally. We then categorized vowels into numbers as follows: *a*(1), *i*(2), *u*(3), *e*(4), *o*(5), *n*(6), and *x*(7). To analyze the audio data, we imported the audio file into a software (Audacity: version 2.0, The Audacity Team), played the voice at each frame (25 FPS), judged how the voice sounded, and coded with the above categories by coders. If there was no voice, we categorized the frame into *no-voice* (0); if there was a voice, we categorized it according to each vowel, a syllabic nasal, or an assimilated sound as described above (1, 2, 3, 4, 5, 6, 7). After categorization, we obtained two categorical data: first, sequential data of seven categories without any time information, and, second, time series data that included temporal information (i.e., a time stamp at 25 Hz) using eight categories from 0 to 7, as shown above.

The *joint recurrence analysis* was used to analyze two physically different time series (Marwan et al., 2007). A joint recurrence point can be considered as joint probability in which both systems have simultaneous recurrence points (more detail in Marwan et al., 2007). A *joint recurrence plot* (JRP) is a graph that shows all those times at which a recurrence in one dynamical system occurs simultaneously with a recurrence in a second dynamical system. In other words, the JRP is the Hadamard product of the recurrence plot of two systems (Marwan et al., 2007). That means only recurrence points contained in *both* RPs keep being plotted (i.e., If*RP_Ai,j_* = 1 *and RP_Bi,j_* = 1, *then JRP_ABi,j_* = 1,*i,j* = 1,,*N*), but in other cases which one or none of both RPs recur, no recurrence points appear (i.e., If*RP_Ai,j_* = 1 *and RP_Bi,j_* = 0, *or RP_Ai,j_* = 0 *and RP_Bi,j_* = 1, *or RP_Ai,j_* = 0 *and RP_Bi,j_* = 0, *then JRP_ABi,j_* = 0, *i,j* = 1,,*N*). JRPs capture the commonalities between two systems (i.e., signals or time series) as coinciding instances of recurrence between the individual RPs of those systems (Wallot et al., 2016). First, each RP is constructed for each system, then their JRP can be computed by joining the plots together, so that common instances of recurrences are kept, but different instances between the two RPs are discarded (Wallot et al., 2016). Joint RQA (JRQA) measures such as *RR* and *maxL* as explained above (in Data Analysis) can be calculated from the JRP in the same way as auto/cross RQA. Originally, the joint method was proposed for two continuous time series, which can recur simultaneously in their individually reconstructed phase spaces, to compare two physically different systems at different units or dimensions. We extended this to compare continuous (motion) data with categorical (rap) data. Thus, the joint method extracts JRQA measures from JRPs by preserving only the points recurring in both categorical and continuous plots.

We conducted a surrogate data test using a random shuffle data set (Kantz and Schreiber, 2003), to confirm that RQA measures calculated from our original data set deviated from an underlying baseline (randomized) distribution. By comparing output measures from the original data set with those from the randomly shuffled surrogate data set under same input parameter settings, we can statistically verify that RQA measures are not consistent with the trends in this baseline (Shockley et al., 2003; Athreya et al., 2014).

We performed recurrence analyses using the MATLAB toolbox “CRP TOOLBOX,” version 5.22 (Marwan and Kurths, 2002), and the R package “crqa,” version 1.0.9 (Coco and Dale, 2014). We determined the optimal values for input parameters with reference to the standard guidelines for the RQA method (Webber and Zbilut, 2005; Marwan et al., 2007) using *average mutual information* (Fraser and Swinney, 1986) for determining the delay (i.e., finding the first minimum in mutual information) and *false nearest neighbor method* (Kennel et al., 1992) for determining the dimension by increasing it in integer steps until the recruitment of nearest neighbors becomes unchanging (Webber and Zbilut, 2005). The radius was chosen so that any RR values in the dataset should not be zero or too high percent and also should obtain robust results (for more details, see Webber and Zbilut, 2005). As a result, for continuous data, we chose parameters of 10 for time delay, 3 for embedding dimensions, and 0.75 for the radius with *z-score* normalization, while for categorical data, we input 1 for time delay and embedding dimensions, and 0.001 for the radius. To align the data lengths between categorical and continuous time series, we removed last 20 points from categorical data because 20 points were lost from continuous data by embedding it into the three-dimension (*m* = 3) phase space consisted of original and two10 delayed (*d* = 10) time series (i.e., (*m*− 1)^∗^*d* = 20).

## Results

### Categorical Recurrence Plot: Rap Data

**FIGURE 2:**
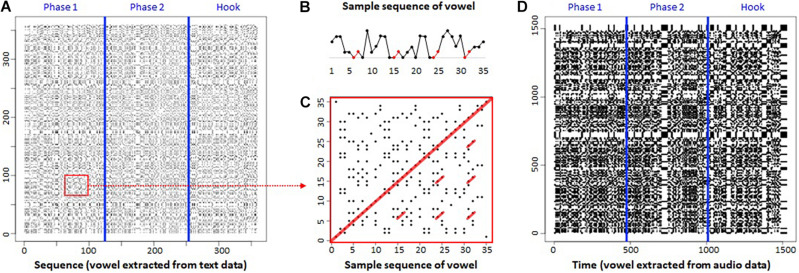
Categorical recurrence plot (CaRP) of rap. **(A)** Standard CaRP. **(B)** Sample sequence of vowel. **(C)** Part of CaRP. **(D)** Proposed CaRP. Both *x*- and *y*-axes represent sequence or time series.

Figure 2A shows the categorical RP (CaRP) of the lyrics of the current rap song generated by the standard procedure (with neither temporal information nor a time stamp). Here, we report the partial result of analysis of the tune, the first Verse and the Hook. We indicated three phases consisting of the first part of the Verse (Phase 1), the latter part of the Verse (Phase 2), and the Hook by adding two blue lines (see Figure 2A). Using vowels as a unit of analysis, the lyric consisted of 359 units (Phase 1: 124, Phase 2: 129, Hook: 106). The CaRP does not exhibit a random dot distribution, but a structured pattern across the phases.

Figure 2B presents a sample sequence of vowel units, while Figure 2C shows its CaRP, extracted from Figure 2A (red square). Red circle markers in Figure 2B indicate repetition (i.e., rhyming) of the same vowel units (i.e., *a*–*i*) four times in part of the lyric. The same part appears in Figure 2C as red lines parallel to the diagonal line in the center of CaRP. These parallel diagonal line structures can be interpreted as a rhyming structure, which appeared temporally. These results illustrate that CaRP can provide a visualization of rhyming structure in musical lyrics.

Figure 2D presents the proposed transformation on CaRP that contains temporal information (i.e., a time stamp at 25 Hz). It has 1,527 points (25 Hz, approximately 60 s) including vowels and a no-voice zero value. Accordingly, it is possible that the same value (e.g., “*a*”) can appear successively; for example, “*a*” can repeat 25 times if the voice stays for 1 s. By adding such temporal information as a time stamp, we integrated categorical data with continuous data within the same framework (joint recurrence analysis), as discussed below. At a glance, this proposed transformation appears to reveal more obvious structured patterns than the standard method, comparing Figures 2A,D. For example, the transition point where the phase changed, or which was a *break* and *pause* in the tune, can be observed as a white band that indicates a no-voice state (Figure 2D). These characteristics seem to express the original music (rap performance) and its temporal structure more clearly.

Our results show that CaRPs can extract a repetitive structure or recurrence pattern of the lyric and rap performance. The proposed method can visualize the RPs in a more informative way by including temporal information.

### Continuous Recurrence Plot: Motion Data

**FIGURE 3:**
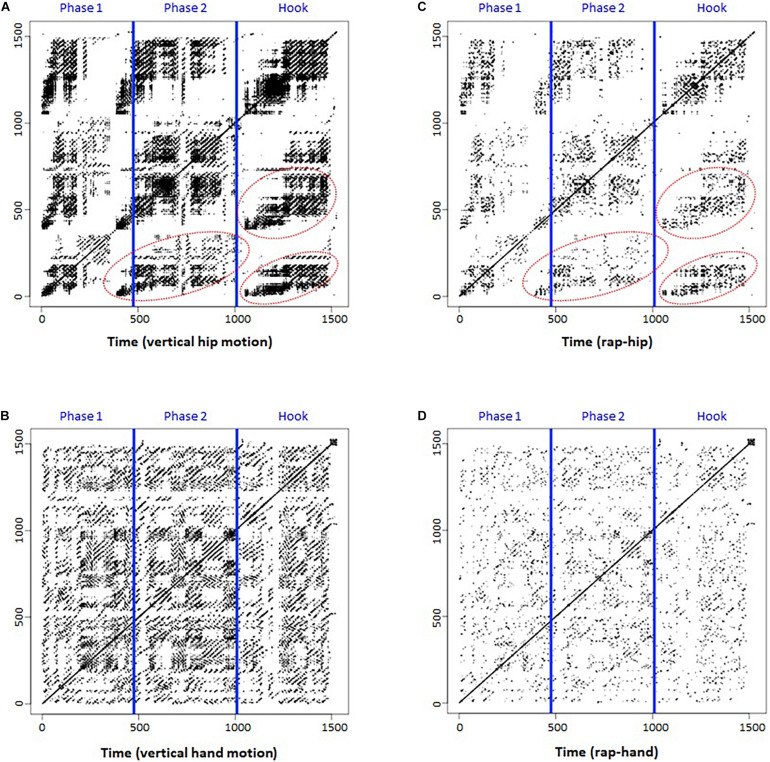
Continuous recurrence plot (CoRP) of rap and Joint recurrence plot (JRP). **(A)** CoRP of hip. **(B)** CoRP of hand. **(C)** JRP of rap-hip. **(D)** JRP of rap-hand. Both *x*- and *y*-axes represent time series.

Figure 3A represents the continuous RP (CoRP) of hip motion in the vertical direction. Blue lines separate the phases again. We assumed that the vertical hip motion could represent whole-body rhythm. The CoRP shows a recurrence pattern at the macro level, with clearly exhibited patterns that deviate from a random dot distribution of an RP based on randomized data. In each phase, recurrence points are shown as a whole-body beat rhythm repeatedly. Furthermore, a similar recurrence structure can be found in the red areas (i.e., Phase 1-Phase 2, Phase 1-Hook, and Phase 2-Hook). These results suggest that the participant beat out a rhythm with whole-body movement and that similar/common rhythm patterns can be found across the phases.

Figure 3B shows the CoRP of hand (i.e., right wrist) motion in the vertical direction. Blue lines separate the phases again. We chose the right wrist marker motion for analysis, because the participant was right-handed and showed specific hand movements, such as beating or gesturing, during the rap performance. Compared with hip motion, hand motion seemed to be more closely related to rap performance and to have a high frequency. As a result, its RP (Figure 3B) shows a more detailed recurrence pattern at the micro level than that in Figure 3A.

### Joint Recurrence Plot

Figures 3C,D depict the joint RP (JRP) of rap-hip coordination and rap-hand (i.e., right wrist) coordination. Blue lines separate the phases again. Compared to the CoRP of hip motion (Figure 3A), the JRP of rap-hip coordination seems to hold a common recurrence pattern at the macro level (red circles in Figure 3C). This suggests that the whole-body rhythm was coupled with rap rhythm. Similarly, the JRP of rap-hand coordination (Figure 3D) seems to hold a common recurrence pattern with the RP of hand motion at the micro level (Figure 3B). This can also be considered rap-hand coupling. Comparisons between Figures 3A,C, and between Figures 3B,D provided an impression that the latter plots (JRPs) look like the attenuated versions of the former plots (Continuous RPs). This means that the JRPs hold only the plots which both rap and motion recur and the other plots of each RP (i.e., rap and motion) disappear because the plot shown in only one of each RP is removed by conducting joint method. These results indicate that JRPs can visualize speech-motion coupling during rap performance.

### Recurrence Quantification Analysis

Table 1 shows the results of RQA. It presents the RQA measures (*RR*, *DET*, *maxL* and *L*) of rap (standard and proposed), motion (hip and hand), and joint (rap-hip and rap-hand) calculated by categorical, continuous and joint RQA, respectively. It also provides RQA measures’ values (from minimum to maximum values) obtained from surrogate data test under each original measure shown in gray rows.

**TABLE 1:** Recurrence quantification analysis measures.

		Rap	Rap	Hip	Hand	Joint	Joint
		Standard	Proposed	Vertical	Vertical	Rap-hip	Rap-hand
RR	Original	19.68	17.22	7.91	3.77	1.68	0.79
	Surrogate	19.68–19.68	17.22–17.22	5.07–5.57	3.27–3.41	0.86–0.96	0.55–0.58
DET	Original	36.2	91.85	94.23	76.89	76.84	61.35
	Surrogate	34.46–35.89	31.01–31.50	9.17–11.08	6.06–7.07	1.61–2.22	0.77–1.39
maxL	Original	18	60	435	229	16	35
	Surrogate	5–8	7–9	4–5	3–5	2–3	2–3
L	Original	2.28	3.74	4.35	2.88	2.82	2.66
	Surrogate	2.21–2.27	2.20–2.21	2.05–2.07	2.03–2.05	2.00–2.03	2.00–2.03

**The surrogate columns show ranges from the minimum to the maximum value in each surrogate data set (Min–Max).**

#### Categorical RQA

The proposed method provided higher values in *DET*, *maxL* and *L* than the standard method. This came as a result of adding temporal information at 25 Hz, because it can realize successive values. In particular, higher values in these diagonal-related measures (*DET*, *maxL* and *L*) suggest that the proposed method can extract more real temporal structure of the lyrics.

#### Continuous RQA

The total hip RQA measures were higher than hand RQA measures. These results suggest that the participant maintained a stable whole-body rhythm, although he moved his dominant hand rhythmically, but in a complicated manner, synchronizing with the rap lyric and beat during rap performance. To address this possibility, the relationship between hand movement (e.g., gesture) and rap lyrics can be researched in more detail in future studies of this particular kind of performance. It is added here only as an intriguing possibility.

#### Joint RQA

The joint method extracts JRQA measures from JRP by preserving only the points recurring in both categorical and continuous plots. Therefore, JRQA measures indicate the co-occurring or co-varying features of two fundamentally different types of data, which cannot be extracted by either individual method alone (i.e., categorical or continuous). Comparing the JRQA results with each RQA result (rap itself or motion itself) showed that the former measures were lower than the latter measures because the joint method preserve only the point where both time series recurred. The diagonal-related measures (*DET*, *maxL* and *L*) again remained high, suggesting that the current joint method could extract temporal structure of rap performance in terms of speech-motion coupling. While *RR* and *DET* were higher in rap-hip coordination than in rap-hand coordination, interestingly, *maxL* was higher in rap-hand coordination than in rap-hip coordination. This suggests that hand movement is likely to couple with rap performance more sustainably and is involved in the content of the lyrics. We found that the right hand of the participant seemed to express the lyric contents, match with the rap tempo (e.g., beating rhythm) and correlate with rapping. Again, these are only promissory observations. The main illustration with this analysis, however, is that calculating an informative JRP is possible under the proposed CaRP.

#### Random Shuffle Surrogate Data Test

To confirm whether observed recurrence patterns are artifacts or not, we conducted the random shuffle surrogate data test (Kantz and Schreiber, 2003). Results revealed that RQA measures of rap data without *RR* were significantly higher in the original data set than the randomly shuffled data set. In the case of *RR*, because the categorical data of both original and shuffled data preserve the same set of data (i.e., vowels in the case of our data), the values (*RR*) should be the same between the original and surrogate data. On the other hand, in the case of output measures of continuous motion data and joint RQA, all measures were significantly higher in the original data set than the randomly shuffled data set. This surrogate analysis verifies that input parameters of RQA were appropriate, and that observed measures of RQA from the original data were not contained inside the distribution generated from randomization (Shockley et al., 2003; Athreya et al., 2014).

Results of Joint RP and RQA showed a possibility to visualize and quantify speech-motion coupling calculated from different kinds of data, e.g., verbal (categorical) and non-verbal (continuous), by using joint recurrence analysis method to integrate categorical data with continuous data. Comparing RQA measures between original and surrogate data supports the validity of our proposed method.

## Discussion

In this paper, we introduced temporal information to the standard categorical recurrence analysis. To do this, we assigned a timestamp to symbolic codes for the rap performance. Although some previous studies have applied similar methods by adding timestamps to verbal and nonverbal or musical behaviors using recurrence analysis (Louwerse et al., 2012; Walton et al., 2018), they analyzed only categorical data or analyzed categorical and continuous data separately. The present study, however, aligned categorical and continuous time series to study the dynamics of complex human behavior in an integrated way. This alignment then permits calculation of joint recurrence analyses. Our single-case illustration shows that this approach may reveal the lyrical structure and the temporal structure (i.e., rhythm) of rapping (singing) or beat (music) itself more clearly. This is may be because the proposed timestamp assignment expands or contracts meaningful variation in the performance itself. Furthermore, we applied the joint recurrence method to integrate categorical data (rap) with continuous data (bodily motion). By employing such integration, we showed the applicability of the joint recurrence method to the investigation of the speech-motion coordination/coupling and suggested the possibility of visualizing and quantifying it.

Our current pilot study focused on hip-hop music, a music genre that has a relatively obvious rhythm and a repetitive/recurring structure (i.e., rhyme) in its lyrics, which helped us to investigate speech-motion relationship. We guessed that this relationship would be relatively easy to extract using the joint RP and RQA. Some similarities between rap dynamics and motion dynamics were found because common auditory information (i.e., a musical track) might affect these dynamics. In future studies, musical data can be transferred to time series by previous study’s method which used the key or note data (Walton et al., 2018). Then researchers can investigate the coordination/coupling relationship among lyrics, body movement and music. For gestural expression, by adopting a detailed coding used in previous study (e.g., Louwerse et al., 2012), we can obtain further insights on how performers express their lyrical and artistical contents. For linguistic features, although the present study focused on temporal information (i.e., rhythmic structure of vowels), researchers might also examine contents or meanings of lyrics by applying the conceptual RQA method (Angus et al., 2012; Tolston et al., 2019).Moreover, further investigation on *when* JRQA measures increase (i.e., co-occurring or co-varying point) and *what* happens (e.g., lyrics, gesture) at the moment within both lyrical and movement domains, would be needed to cultivate a deeper understanding of the performance.

As a methodological illustration, we analyzed only one performance in this study. Follow-up analysis is thus needed to confirm whether our specific findings are robust by collecting and analyzing further data. In application of this methodology to a much larger dataset, it would be possible to compare original data to virtual pair data of rap-motion coupling generated from other rappers’ performance data. This analysis would show that the current result was not produced by an artifact or possible random matching in terms of surrogate data method (Shockley et al., 2003). It would broaden the applicability of the joint method that integrates categorical data (rap) with continuous data (motion).

Although the present study focused on an intrapersonal coordination between speech and motion, interpersonal coordination across participants can also be examined within the same framework as investigated by previous studies that have applied the recurrence analysis to various joint action tasks (e.g., Fusaroli et al., 2014; Shockley and Riley, 2015).

The proposed method could be applied to not only ready-made songs but also improvisational freestyle performance, including various music genres. Improvisational performance is more like everyday social interaction, in the sense that it also has complex aspects emerging from real-time interaction (Walton et al., 2018). The dynamical methods (e.g., recurrence analysis) are also expected to reveal the creative process in detail using more advanced techniques (e.g., the windowed sliding method; Coco and Dale, 2014; Kodama et al., 2018). We also aim to apply the framework not only to experimental situations but also to more ecological situations, such as the practical field of artistic performance, natural daily interaction (D’Ausilio et al., 2015; Dale, 2015; Sekine and Kita, 2015; Shimizu and Okada, 2018) and clinical interaction in psychological and medical fields (Zivotofsky and Hausdorff, 2007; Nagaoka and Komori, 2008; Ramseyer and Tschacher, 2011; Goldstein et al., 2020) involving speech-motion coordination in the future.

## Data Availability Statement

The original contributions presented in the study are included in the article/Supplementary Material, further inquiries can be directed to the corresponding author/s.

## Ethics Statement

The studies involving human participants were reviewed and approved by the Research Ethics Committee of the Kanagawa University. The patients/participants provided their written informed consent to participate in this study.

## Author Contributions

KK, DS, and KS designed this study, acquired and analyzed the data, and wrote the manuscript. RD contributed to data analysis and manuscript drafting. All authors have read and approved the final manuscript.

## Conflict of Interest

The authors declare that the research was conducted in the absence of any commercial or financial relationships that could be construed as a potential conflict of interest.
