# Supplementary material for: An Approach to Aligning Categorical and Continuous Time Series for Studying the Dynamics of Complex Human Behavior
Source: Front Psychol. 2021 Apr 16;12:614431. doi: 10.3389/fpsyg.2021.614431 (PMC8085256; doi:10.3389/fpsyg.2021.614431)
Supplement: Supplementary file 2 [file Table_2.docx]

# Parameter values are from our study; any use of this method should consult the previous studies (e.g., Coco & Dale, 2014; Webber & Zbilut, 2005).

# Our data (e.g., rap and hip) are available in the supplementary materials.

install.packages('crqa')

library('crqa')

setwd("/Users/your_directory")

# categorical data

cate_data = read.csv("rap.csv", header=F)

# align the data length to the continuous data length [e.g., cut the last 20 points that are lost by embedding the continuous data (N=1547-20=1527)]

# lost data length: (embed-1)*delay [e.g., (3-1)*10=20]

cate_data2 = cate_data[1:1527, 1]

# auto recurrence

cate_res = crqa(cate_data2, cate_data2, delay = 1, embed = 1, rescale = 1,

radius = 0.001, normalize = 0, mindiagline = 2, minvertline = 2,

tw = 1, whiteline = FALSE, recpt = FALSE, side = "both",

method = 'rqa', metric = 'euclidean', datatype = "categorical")

RP_cate = cate_res$RP

cate_res[1:9]

# plot RP

plot_rp = function(RP, xlab = 'i', ylab = 'j', cex = .2) {

if (!is.matrix(RP)) { RP = as.matrix(RP) }

ij = which(RP == 1, arr.ind = T)

plot(ij[, 1], ij[, 2], cex = cex, xlab = xlab, ylab = ylab, pch = 16)

}

plot_rp(RP_cate)

# continuous data

cont_data = read.csv("hip.csv", header=F);

# auto recurrence

cont_res = crqa(cont_data$V1, cont_data$V1, delay = 10, embed = 3, rescale = 0,

radius = 0.75, normalize = 2, mindiagline = 2, minvertline = 2,

tw = 1, whiteline = FALSE, recpt = FALSE, side = "both",

method = 'rqa', metric = 'euclidean', datatype ="continuous")

RP_cont cont_res$RP

cont_res[1:9]

# plot RP

plot_rp(RP_cont)

# JRP = simply the multiplication of the two Recurrence Plots (RPs)

JRP = (RP_cont*RP_cate)

JRP_res = crqa(JRP, delay = 1, embed = 1, rescale = 0, radius = 5, normalize = 0,

minvertline = 2, mindiagline = 2, tw = 1, whiteline = F, recpt = T)

JRP_res[1:9]

plot_rp(JRP_res$RP)
